# Durability of solvent-free one-step self-etch adhesive under simulated intrapulpal pressure

**DOI:** 10.4317/jced.52307

**Published:** 2015-10-01

**Authors:** Shaymaa M. Nagi

**Affiliations:** 1 Restorative and Dental Materials Research department, National Research Centre, Giza. Egypt

## Abstract

**Background:**

There are different solvents presented in simplified adhesives. Bond-1 SF has been developed, which contains neither water nor organic solvents, in order to eliminate technical issues in terms of evaporation of solvents and concerns for the durability of resin-dentin bond. Thus this study was conducted to evaluate the microtensile bond strength (?TBS) of solvent-free and ethanol-based one-step self-etch adhesives to dentin under simulated intrapulpal pressure (IPP).

**Material and Methods:**

Occlusal surfaces of human molars were prepared to expose mid-dentin depth. Bond-1SF Solvent-Free SE [SF] and AdperTM easy one adhesives [AE] were applied on dentin specimens. Resin composite build up was done in increments. Then specimens were stored under simulated IPP 20 mmHg, immersed in artificial saliva at 37 ºC for 24 hours (24h) and 6 months (6m). Specimens were sectioned into sticks of (1 mm²) to be tested for (?TBS) using a universal testing machine. Both fractured sections of each stick were inspected using a stereomicroscope at 40× magnification to determine the mode of failure. Data were statistically analyzed by Two-way ANOVA of Variance.

**Results:**

There was no statistically significant difference between the mean ?TBS of both [SF] and [AE] adhesives at both aging periods, 24h and 6m (*p*< 0.1103) and (*p*< 0.7148) respectively. Only for [AE] there was statistical significance for aging periods (*p*< 0.0057*). The most represented modes of failure were adhesive failure at tooth side.

**Conclusions:**

Under simulated IPP solvent-free adhesive [SF] had comparable performance as ethanol-based adhesive [AE] when bonded to dentin substrate.

** Key words:**Bond strength, dentin, simulated intrapulpal pressure, self-etch adhesives, solvents.

## Introduction

The improvement of adhesive dentistry throughout the life of dentistry makes bonding to tooth structure more simplified. This simplification was done to reduce the steps of clinical application, technique-sensitivity and material-related factors that affect bond strength to tooth structure (1).

One of the greatest challenges in adhesion is related to the need of dentin being slightly moist before being properly bonded. Wa-ter is an essential component of dentin matrix to prevent the collapse of the collagen network after etch step. However, excessive moisture can adversely affect hybrid layer durability due to degradation of either collagen fibrils or resin material (2). Different solvents presented in simplified bonding agents are responsible for either carrying excess water out or infiltrating resin monomers into interfibrilar dentin. However, solvents must be eliminated after having completed their function (3), as it has been demonstrated that the residual contents of the solvents in the adhesives become a critically important factor in predicting the bond inte-grity or the longevity of adhesive resins (4).

A unique one-step adhesive system, Bond 1 SF (Pentron Clinical, California, USA) has been developed, which contains neither water nor organic solvents in the ingredients in order to eliminate technical issues in terms of evaporation of solvents and concerns for the durability of resin-dentin bond.

However, there is little information on its bonding performance. Moreover no studies tested the bond strength of solvent free-adhesive to dentin under the challenge of some in vivo simulating conditions. Thus it seems to be of value to evaluate the microtensile bond strength of solvent free-adhesive to dentin under the challenge of some in vivo simulating conditions in terms of (intrapulpal pressure simulation, immersed in artificial saliva at 37 ºC) compared to that of ethanol- based one step self-etch adhesive at different aging periods.

## Material and Methods

-Selection and grouping of teeth:

Sixty recently extracted sound human third molars from 18-28 year-old patients scheduled for extraction were collected. An approval from the National Research Centre Medical ethics committee, Egypt 2003 was taken for using dentin dental tissue in this study. The teeth were then stored in phosphate buffer solution (g/L): [(Na2HPO4 (0.578), KH2PO4 (0.353) dissolved in distilled water containing 0.02% sodium azide] adjusted at pH=7, and stored at 4?C for a maximum periods of one month before being used (5). Teeth were divided into two main groups, (n=30) according to the adhesive systems utilized; [Ethanol-based one-step self-etch adhesive] AdperTM easy one [AE], that was used with FiltekTM Z350 resin composite, and [Solvent-free one-step self-etch adhesive] Bond-1SF Solvent-Free SE adhesive [SF], that was used with Alert condensable composite. Then each group was divided into 2 subgroups (n=15) according to aging periods 24h and 6m. Materials names, composition and manufactures and batch number were presented in  Table 1.

**Table 1:**
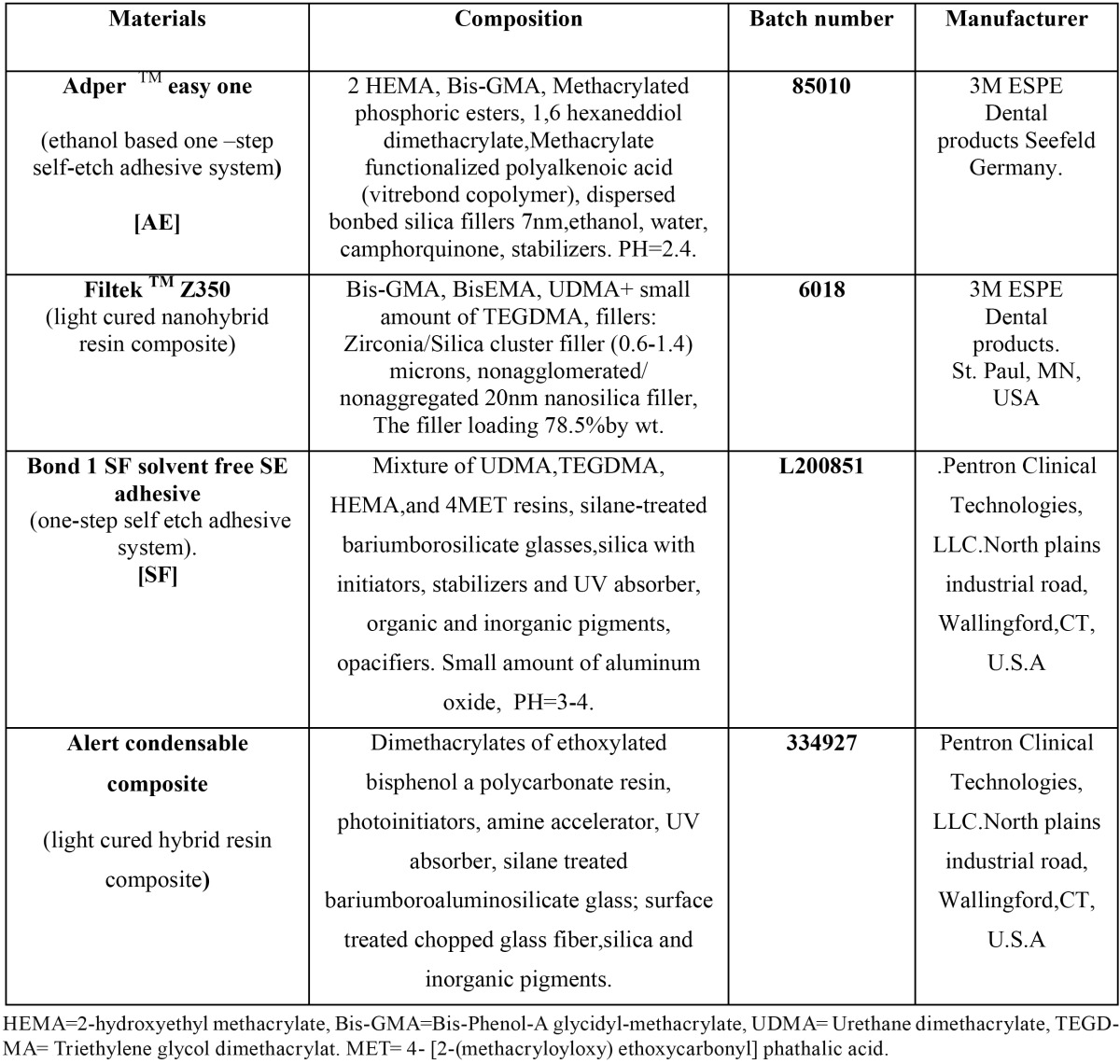
Material names, composition, batch numbers and manufacturers.

-Specimens preparation.

Roots of all teeth were cut off 2mm gingival to cemento-enamel junction. Then the pulp tissue was carefully removed with excavator (Dentsply/Maillefer, Ballaigues, Switzerland) avoiding contact with the walls of the pulp chamber (6). Then occlusal enamel was grinded to expose mid-coronal dentin. The height of the remaining dentin available for bonding was measured using precise caliper. Only crown segments with a remaining dentin thickness of 2 mm were used in this study (7). Then the grinded dentin surfaces were finished for one minute by wet grinding with a 600-grit SiC paper to achieve a standardized smear layer (8). Prepa-red crown segment was then centrally glued to a teflon plate (150mm diameter x 1mm thickness), then a butterfly stainless steel needle gauge 19 (Shanchuan Medical Instruments.Co.ltd, Zibo, China) was inserted and tightly fit to a central hole at the center of the Teflon plate. Centered crown and the butterfly needle were embedded in chemically cured polyester resin (Polyester resin #2121, Hsein,Taiwan ) till 1mm gingival to the CEJ. All specimens were connected to the intrapulpal pressure assembly adjusted at 20 mmHg pressure for 24h before the restorative procedures to simulate the intraoral environmental condition and to keep the teeth wet before bonding (6,9).

-Restorative procedures:

During restorative procedures the intrapulpal pressure was reduced to 0-5 mmHg simulating the intrapulpal pressure after taking local anesthesia with vasoconstrictor (6,10). Adhesive systems were applied according to the manufactures’ instructions. Corres-ponding resin composite of each adhesive system was applied in two increments 1.5 mm each, then each increment was light cured for 40 seconds using bluephase C5 light curing unit (Ivoclar Vivadent, Schaan, Leichtenstein) at an intensity ? 500 mW/cm2 to form a resin composite block of approximately 3mm height. After resin composite application, the intrapulpal pressure was adjusted to 20mmHg and all the specimens were immersed in artificial saliva (11). Then, all specimens were incubated at 37?C for 24h and 6m.

-Microtensile bond strength (?TBS) testing:

All the bonded specimens were sectioned into multiple sticks, of cross sectional surface area of approximately (1±0.05) mm². For standardization, only sticks of the same cross sectional area, length and remaining dentin thicknesses were included in the study (n=24?class), using a precise caliber (Tresna Measuring Instrument, TDS-150, Germany). Sticks were stressed in tension using universal Lloyd testing machine (Lloyd instruments Ltd, an Ametek company, UK) travelling at a cross-head speed of 0.5 mm/minute until failure. Microtensile bond strength (MPa) values were determined by computing the ratio of maximum load (N) by the bonded surface area in mm2.

-Fractographic analysis:

Both fractured sections of each stick (tooth side and resin composite side) were inspected using stereomicroscope (Stereomicros-cope Nikon SMZ-10-Japan) at 40× magnification to determine the mode of failure. Failure mode was categorized either into:

Type 1: Adhesive failure at tooth side, Type 2: Cohesive failure in adhesive layer, Type 3: Mixed failure (adhesive failure at tooth side / cohesive in adhesive layer).

-Statistical analysis.

Results of microtensile bond strength values, and mode of failure were recorded and tabulated. Initially Two-way analysis of variance ANOVA was done to detect effect of each variable (adhesive systems, and aging periods) on the microtensile bond strength values as well as their interaction. Then one-way ANOVA was used, followed by Pair-wise Newman-Keuls post-hoc test to detect the significance between subgroups. Student t- test was used to compare between aging periods. Statistical analysis was done using graph Pad person four statistical analysis soft ware for windos *P* ? 0.05 considered significant at all tests.

## Results

**Figure 1:**
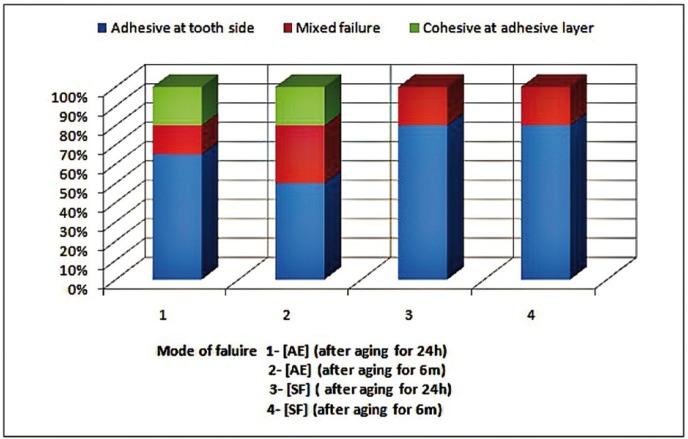
Percentage mode of failure.

 Table 2 represents dentin ?TBS (Mean ± SDs) of the tested adhesive systems at 24h and 6m aging periods. Two-way ANOVA revealed statistical non significance for each studied variable (adhesive systems, and aging periods) as well as their interaction. Except for [AE] there was statistical significance for aging periods as indicated by ANOVA test *p*=0.0057*. For [AE]; recorded higher statistically non significant µTBS mean value compared to [SF] at both aging periods 24 hours and 6 months as indicated by ANOVA test, *p*=(0.1103) and *p*=(0.7148) respectively. The most represented modes of failure were adhesive failure at tooth side for both [AE] and [SF] as shown in figure 1.

**Table 2:**
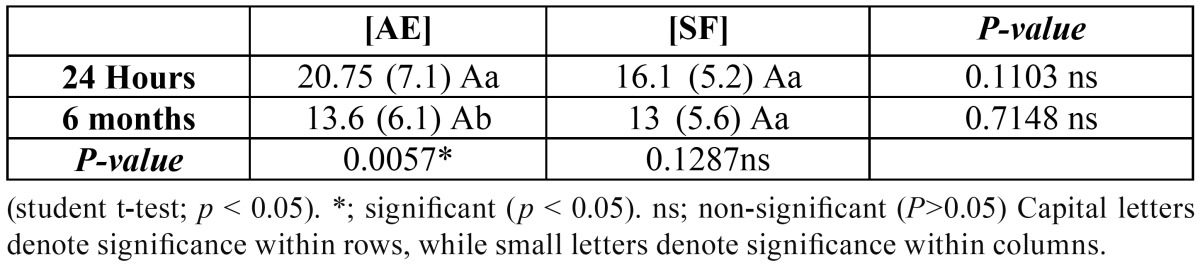
Dentin microtensile bond strength (Mean ± SDs) of the tested adhesive systems at 24 hours and 6 months aging periods.

Discussion

After 24 hours and six months aging under intrapulpal pressure simulation, artificial saliva immersion at 37ºC, there was no statistical significant difference between the mean ?TBS of [AE] and [SF]. This might be due to the relatively comparable near high pH values of [AE] (PH=2.4) and [SF] (PH=3). Both adhesives were considered as mild self-etch adhesives, and their same mechanism of interaction with dentin was limited to a few hundreds of nanometers, which produce intense intertubular microporosity with residual smear layer and preservation of smear plug in dentin (12-16).

The immediate bond strength values do not always correlate with the long term bond stability, since degradation throughout the bonded interface does not occur rapidly (17). This was clearly seen with the ?TBS of [AE] as its bond strength decreased significantly after aging for 6m under intrapulpal pressure simulation and artificial saliva immersion at 37°C. The reasons for the inadequate performance might be due to combination of acidic, hydrophilic and hydrophobic monomers along with organic solvents and water in a single bottle. This was responsible for the high hydrophilicity of these systems. So they were more prone to water sorption and subsequent reduction in the mechanical properties. Consequently they were considered semi-permeable membranes to water diffusion from the underlying dentin across the adhesive layer creating water-filled channels within the adhesive. Alt-hough this might not have any effect of the immediate resin-dentin bonds, it is likely that it plays a deformative role on the water uptake along time (18-19). Also the high concentration of HEMA has been recently recognized to lower vapor pressure of water and so prevent its complete removal from the adhesive during bonding and promote water to be bonded in an unstable soft hydrogels within both hybrid and adhesive layers (17-19). Beside, there was a differential infiltration gradient established as a consequence of phase separation within the adhesive, and due to differences in molecular weight or affinity to dentin of the infiltrating compounds of the adhesive system (20).

Another reason was, a relatively high concentration of ethanol solvent is required to keep these adhesives blended in solution, and air drying is not able to accomplish significant solvent evaporation (21). Residual solvents produce localized areas of incomplete monomer polymerization, generating porosities within the bonded interfaces that, in turn, may permit inward diffusion of oral fluids, lower the conversion of monomers into polymers and increase the water sorption, resulting in lower mechanical properties of the adhesive polymers and hydrolysis of resin and collagen fibrils. Less stiff resin might lead to weaker bonding to dentin (22). Moreover air drying in solvent containing one-step adhesives to enable evaporation of the solvents, makes the bonding layer thinner, which might lead to incomplete polymerization due to the existence of oxygen. Oxygen inhibits the free radical polymerization, resulting in a layer of not or partially polymerized resin. This may result in suboptimal mechanical properties of the adhesive layer and accelerated degradation of the adhesive.

Results of mode of failure of the fractured specimen of [AE] showed high percentage of type 2 failure (cohesive failure in the adhesive layer) and mixed failure after 6m aging which suggested high degree of degradation (18). While regarding [SF] there was no statistically significant decrease in the mean bond strength after aging for 6m under intrapulpal pressure simulation and artificial saliva immersion at 37°C. This might be due to the unique composition of this adhesive. Which contains neither water nor organic solvents in the ingredients in order to eliminate technical issues in terms of evaporation of solvents and concerns for the durability of resin-dentin bond as discussed above. This was supported by the finding of mode of failure of the fractured specimens of [SF] adhesive which showed that the predominant failure were mixed and adhesive at tooth side after 6m aging.

## Conclusions

Solvent free self-etch adhesive Bond1 SF [SF]; revealed adequate and comparable bond strength compared to ethanol-based one-step self-etch adhesives to dentin substrate under simulated intrapulpal pressure.
